# Prospective Associations of Coronary Heart Disease Loci in African Americans Using the MetaboChip: The PAGE Study

**DOI:** 10.1371/journal.pone.0113203

**Published:** 2014-12-26

**Authors:** Nora Franceschini, Yijuan Hu, Alex P. Reiner, Steven Buyske, Mike Nalls, Lisa R. Yanek, Yun Li, Lucia A. Hindorff, Shelley A. Cole, Barbara V. Howard, Jeanette M. Stafford, Cara L. Carty, Praveen Sethupathy, Lisa W. Martin, Dan-Yu Lin, Karen C. Johnson, Lewis C. Becker, Kari E. North, Abbas Dehghan, Joshua C. Bis, Yongmei Liu, Philip Greenland, JoAnn E. Manson, Nobuyo Maeda, Melissa Garcia, Tamara B. Harris, Diane M. Becker, Christopher O'Donnell, Gerardo Heiss, Charles Kooperberg, Eric Boerwinkle

**Affiliations:** 1 Gillings School of Global Public Health, University of North Carolina, Chapel Hill, North Carolina, United States of America; 2 Department of Biostatistics and Bioinformatics, Emory University, Atlanta, Georgia, United States of America; 3 Department of Epidemiology, University of Washington, Seattle, Washington, United States of America; 4 Department of Statistics & Biostatistics, Rutgers University, Piscataway, New Jersey, United States of America; 5 Laboratory of Neurogenetics, National Institute on Aging, NIH, Bethesda, Maryland, United States of America; 6 Division of General Internal Medicine, Department of Medicine, The Johns Hopkins University School of Medicine, Baltimore, Maryland, United States of America; 7 Department of Biostatistics, University of North Carolina, Chapel Hill, North Carolina, United States of America; 8 Division of Genomic Medicine, National Human Genome Research Institute, NIH, Bethesda, Maryland, United States of America; 9 Department of Genetics, Southwest Foundation for Biomedical Research, San Antonio, Texas, United States of America; 10 MedStar Health Research Institute, Hyattsville, Maryland, United States of America; 11 Department of Biostatistical Sciences, Wake Forest School of Medicine, Winston-Salem, North Carolina, United States of America; 12 Division of Public Health Sciences, Fred Hutchinson Cancer Research Center, Seattle, Washington, United States of America; 13 Department of Genetics Lineberger Comprehensive Cancer Center School of Medicine, University of North Carolina at Chapel Hill, Chapel Hill, North Carolina, United States of America; 14 Cardiovascular Institute, the George Washington University, Washington, D. C., United States of America; 15 Department of Preventive Medicine, University of Tennessee Health Science Center, Memphis, Tennessee, United States of America; 16 UNC Center for Genome Sciences, Chapel Hill, North Carolina, United States of America; 17 Department of Epidemiology, Erasmus University Medical Center, Rotterdam, The Netherlands; 18 Cardiovascular Health Research Unit and Department of Medicine, University of Washington, Seattle, Washington, United States of America; 19 Center for Human Genomics, Department of Epidemiology and Prevention, Wake Forest School of Medicine, Winston Salem, North Carolina, Tennessee, United States of America; 20 Departments of Preventive Medicine and Medicine, Northwestern University, Chicago, Illinois, United States of America; 21 Department of Medicine, Brigham and Women's Hospital, Harvard Medical School, Boston, Massachusetts, United States of America; 22 Department of Pathology and Laboratory Medicine, University of North Carolina, Chapel Hill, North Carolina, United States of America; 23 Laboratory of Epidemiology and Population Sciences, National Institute on Aging, NIH, Bethesda, Maryland, United States of America; 24 Department of Health Policy and Management, Johns Hopkins Bloomberg School of Public Health, Baltimore, Maryland, United States of America; 25 National Heart, Lung and Blood Institute's Framingham Heart Study, Framingham, Massachusetts, United States of America; 26 Human Genetics Center, University of Texas Health Science Center at Houston, Houston, Texas, United States of America; San Francisco Coordinating Center, United States of America

## Abstract

**Background:**

Coronary heart disease (CHD) is a leading cause of morbidity and mortality in African Americans. However, there is a paucity of studies assessing genetic determinants of CHD in African Americans. We examined the association of published variants in CHD loci with incident CHD, attempted to fine map these loci, and characterize novel variants influencing CHD risk in African Americans.

**Methods and Results:**

Up to 8,201 African Americans (including 546 first CHD events) were genotyped using the MetaboChip array in the Atherosclerosis Risk in Communities (ARIC) study and Women's Health Initiative (WHI). We tested associations using Cox proportional hazard models in sex- and study-stratified analyses and combined results using meta-analysis. Among 44 validated CHD loci available in the array, we replicated and fine-mapped the *SORT1* locus, and showed same direction of effects as reported in studies of individuals of European ancestry for SNPs in 22 additional published loci. We also identified a SNP achieving array wide significance (*MYC*: rs2070583, allele frequency 0.02, *P* = 8.1×10^−8^), but the association did not replicate in an additional 8,059 African Americans (577 events) from the WHI, HealthABC and GeneSTAR studies, and in a meta-analysis of 5 cohort studies of European ancestry (24,024 individuals including 1,570 cases of MI and 2,406 cases of CHD) from the CHARGE Consortium.

**Conclusions:**

Our findings suggest that some CHD loci previously identified in individuals of European ancestry may be relevant to incident CHD in African Americans.

## Introduction

The lifetime risk of coronary heart disease (CHD) at age 40 year in the U.S. is 1 in 2 for men and 1 in 3 for women [1], and higher in African Americans than whites largely due to a higher burden of cardiovascular disease (CVD) risk factors in this population [2]. CHD affects an estimated 16.3 million Americans, with a prevalence of 7.9% in African American men and 7.6% in African American women [2]. While in most industrialized countries CHD is the main cause of death in adults, it also is a leading cause of heart failure, which also disproportionally affects African Americans [3]. Genetic susceptibility to CHD has been recently evaluated in genome wide association (GWA) studies, which reveal approximately 44 loci for CHD in individuals of European ancestry [4]–[12] and in East Asians [13]–[16]. Collectively, these variants represent a small fraction of the genetic contribution to CHD risk [12].

Despite the evidence for genetic susceptibility to CHD in African Americans [17], there are few studies of CHD in African American individuals [18], [19] perhaps due to limited availability of genotyped individuals and to the lower representation of African ancestry-specific genetic variants by current GWA arrays. Barbalic *et al.* studied the association of genotyped GWA (500K Affymetrix 6.0) single nucleotide polymorphism (SNPs) with incident CHD in 2,905 African American participants of the Atherosclerosis Risk in Communities (ARIC) study, and replicated a significant SNP association near the *PFK1* gene in 8,000 African American women from the Women's Health Initiative (WHI) [18]. The study by Lettre *et al.* tested the association of genotyped and HapMap imputed GWA SNPs with prevalent CHD. It included 9,119 African Americans from the Candidate Gene Association Resource (CARe) Project, of which 3,269 were ARIC participants [19]. These prior studies included small sample sizes for discovery, had low coverage of CHD GWA genomic regions [18], [19], or studied prevalent CHD [19]. Published CHD loci have not been replicated in African Americans [19]. In our prior study of the Population Architecture using Genomics and Epidemiology (PAGE) study [20], we evaluated the association of the published SNP with incident CHD events in three large cohort studies (ARIC, WHI and the Cardiovascular Health Study [CHS]) by direct genotyping of SNPs [21]. We were able to replicate prospective associations of CHD SNPs in individuals of European ancestry [21] but not in the African American sample, which included 8,018 individuals and 808 CHD events. Differences in linkage disequilibrium (LD) and tagging of functional SNPs in individuals of African ancestry, and differences in allele frequencies or genetic and environmental background across populations may explain much of these findings.

The NHGRI supported PAGE study genotyped African American individuals on the MetaboChip, a high density custom Illumina iSelect array of ∼200,000 SNPs that includes dense genotyping coverage for loci previously associated with atherosclerotic, CVD and metabolic traits [22]. This array allows examining the association of genotyped low frequency variants not previously examined in GWA studies, in addition to have a more comprehensive coverage of previously identified CHD loci compared to arrays used in prior studies. We sought to examine the evidence for association of validated CHD loci identified in case-control studies with incident CHD using a sample of up to 8,200 PAGE African American individuals drawn from two large prospective cohort studies, ARIC and WHI. Additionally, we sought to fine map these CHD susceptibility loci, and characterize novel variants influencing CHD risk in African Americans.

## Material and Methods

All participants included in these analyses have given consent for genetic studies and data sharing. The University of North Carolina IRB and the Atherosclerosis Risk in Communities and Women's Health Initiative Publication and Presentation Committees have approved this study. Patient records/information was anonymized and de-identified prior to analysis.

### African American MetaboChip genotyped PAGE samples

The PAGE study includes eight well characterized population-based studies assembled in four member studies of PAGE and its coordinating center. These are Causal Variants Across the Life Course (CALiCo), which includes the ARIC, Coronary Artery Risk Development in Young Adults (CARDIA), the Cardiovascular Health Study (CHS), the Hispanic Community Health Study/Study of Latinos (SOL), and Strong Heart Study (SHS); Epidemiological Architecture for Genes Linked to Environment (EAGLE), accessing three National Health and Nutrition Examination Surveys (NHANES); the Multiethnic Cohort (MEC) Study; and WHI. All individual-level genotypes and phenotypes are currently publicly available in dbGAP (ARIC is phs000280 - PAGE substudy number is phs000223, and WHI is phs000200 (PAGE substudy number is phs000227). We used data from African American participants of the CALiCo-ARIC and the WHI studies.

The ARIC study is a multi-center cohort and community surveillance investigation in predominantly bi-racial populations (white and African Americans) [23]. ARIC recruited 15,792 individuals (of which 4,266 are African Americans) aged 45–64 years from four communities in Forsyth County, N.C., Jackson, M.S., Minneapolis, M.N., and Washington County, M.D. for a baseline examination in 1987–1989, with four follow-up examinations. Annual follow-up and community surveillance identified CHD events (defined below) which are then classified by an expert panel of physicians based on review of hospital records, death certificates and interviews of next of kin [23]. All participants included in these analyses have given consent for genetic studies and data sharing. WHI is a prospective study investigating post-menopausal women's health in the U.S [24]. A total of 161, 838 women aged 50–79 years old were recruited from 40 US clinical centers between 1993 and 1998 to participate in an observational study (OS) and in three clinical trials (CT). Annual (OS) and semi-annual (CT) follow-up identified self-reported events which were then classified by an expert panel of physicians based on review of hospital records, death certificates and interviews of next of kin [25]. The CHD classification criteria are comparable between the WHI and ARIC and were further harmonized for this study. A subset of 2,200 WHI African American women was selected for the MetaboChip genotyping (sample 1) and an additional 2,809 African American women genotyped in the PAGE extension phase (sample 2) and included in the discovery phase. Women were selected for genotyping on the basis of DNA and biomarker availability, and consent. All participants included in these analyses have given consent for genetic studies and data sharing. Additional study descriptions are shown in S1 File.

### Genotyping and quality control (QC) in PAGE

The MetaboChip array design includes SNPs associated at genome-wide significance to any human trait published in the NHGRI GWA catalog as of August 1, 2009 (including loci identified in the CARDIoGRAM for CHD), and an additional proxy SNP with r^2^>0.90 in the CEU HapMap II dataset, plus up to four additional SNPs with r^2^>0.5 in the YRI HapMap II dataset. SNPs were also selected to fine-map regions of interest related to metabolic traits; copy number variant-tagging SNPs, Major Histocompatibility Complex (MHC) SNPs, SNPs on the X and Y chromosomes, mitochondrial DNA SNPs, and “wildcard” SNPs were also targeted, for a total of approximately 200,000 SNPs. Several validated GWA loci for CHD were fine mapped using 1000 Genome Project (1000 G) genotypes and information from the GWA leading SNP or proxy obtained from large consortia including the CARDIoGRAM, a consortium of case-control studies of CHD [26]. In PAGE, study samples were genotyped separately in WHI and ARIC studies following each genotyping center's standard procedures. HapMap YRI samples were also genotyped to facilitate cross-study QC. Standard QC was applied for samples and SNPs including missing data and Hardy-Weinberg Equilibrium (HWE). We also estimated identity-by-descent (IBD) statistics for all pairs. Principal components were determined using the Eigensoft software separately for each study [27]. We excluded ancestry outliers and first degree relatives. Approximately 161,098 (81.9%) SNPs passed QC filters. The design and performance of this genotyping chip in this African American sample has been described in detail elsewhere [22].

### CHD event definition and follow-up

ARIC and WHI have standardized protocols for the ascertainment and classification of CHD events, defined as acute myocardial infarction (MI) and fatal CHD. In ARIC, CHD events were ascertained from annual follow-up interviews and morbidity and mortality surveillance of communities including hospitalizations and deaths. Events were reviewed by two physicians and differences adjudicated. CHD events were defined as acute hospitalized MI (definitive or probable), definite fatal CHD, or ECG diagnosis of MI. Acute MI was defined based on criteria that included cardiac pain, cardiac markers and ECG readings. Events through December 31^st^, 2007 are included. In WHI, CHD was defined as acute hospitalized MI (definitive or probable) and definite fatal CHD. Acute MI was defined based criteria that included cardiac pain, cardiac markers and ECG readings. Follow-up of events in WHI were through August 2009.

### Replication samples for novel loci and secondary signals in known loci

For novel loci identified in the array wide analyses, we attempted to validate the findings using imputed data from additional studies of African ancestry including WHI (n = 6,265), HealthABC (n = 895) and GeneSTAR (n = 1,059), in addition to 24,024 individuals of European ancestry from the CHARGE Consortium. Because our main findings showed little evidence for heterogeneity across sex-stratified analysis, we allowed for combined sex results for replication. We attempted to replicate secondary signals at known loci in these additional samples and in silico using publicly available meta-analysis data from the CARDIoGRAM plusC4D Consortium (http://www.cardiogramplusc4d.org/downloads/).

A subset of WHI African American women (n = 6,265) were genotyped as part of the SNP Health Association Resource (SHARe) using Affymetrix 6.0 platform. Description of the MetaboChip imputation in this subset is described elsewhere [28]. This sample included 336 CHD incident events.

HealthABC is a prospective cohort study of 3,075 community-dwelling men and women living in Memphis, Tennessee, USA and Pittsburgh, Pennsylvania, USA, aged 70–79 years at recruitment in 1997 [29], of which 42% were African Americans. A random sample of European ancestry and African American Medicare-eligible elders, within designated zip code areas, were contacted. Eligibility included reporting no difficulty with activities of daily living, walking a quarter of a mile, or climbing ten steps without resting. All eligible participants signed a written informed consent, approved by the IRBs at the clinical sites. This study was approved by the IRBs of the clinical sites and the coordinating center (University of California, San Francisco). Individuals with prevalent CHD defined as MI, angina, bypass surgery, or percutaneous transluminal coronary angioplasty (PTCA) procedures or diagnoses reported to the Centers for Medicare and Medicaid Services in the five years prior to baseline, were excluded. Incident CHD was defined as adjudicated event of MI, hospitalization for angina, or adjudicated CHD death in participants who did not have prevalent CHD. The sample included 895 participants and 223 events. Follow-up is through December 31, 2011, for a median of 11.0 years. Genotyping was done on Illumina 1 M microarrays and the GWA data underwent standard QC procedures including evaluation of genotype and self-reported sex concordance, sample call rate of 97% or higher, exclusion of heterogeneity or homozygosity outliers, relatedness (IBD>0.125 were excluded) and ancestry outliers. All SNPs were filtered for inclusion based on a minimum call rate of 97%, HWE>10^−6^, minor allele frequency>1% and a missingness by haplotype p-value>10^−5^. Samples were imputed to the most recent multiethnic panel of the 1000 G reference haplotypes (phase 1 alpha version 3) using MACH and minimac. Analyses were carried out using R v2.14.2, with the first ten principal components in the African American population as a means of controlling for population substructure.

The Genetic Study of Atherosclerosis Risk study (GeneSTAR) study identified probands with documented premature (age <60 years) CHD events including MI, coronary artery bypass surgery (CABG), percutaneous coronary intervention (PCI), or ≥50% stenosis in one or more vessels confirmed on coronary angiography with or without angina symptoms at the time of hospitalization in one of 10 Baltimore, Maryland hospitals. Their apparently healthy siblings <60 years of age and free of known CHD were recruited and screened from 1983 to 2007, and apparently healthy offspring of the probands or siblings were recruited and screened from 2003–2007. Participants were excluded from the study for systemic autoimmune disease, chest radiation exposure, any life-threatening disease (AIDS or advanced cancer), or chronic glucocorticosteroid therapy. Participants completed a standardized health status and cardiovascular disease event questionnaire at approximately five-year intervals after their baseline visit with trained telephone interviewers between 1992 and 2012. For deceased siblings, proxy interviews were completed with the next of kin, and death certificates were obtained. Medical records were then obtained for all reports of a CHD event, any possibly related diagnosis, diagnostic procedure (including exercise tests, thallium imaging, or coronary angiography) or therapeutic procedure (including PCI or CABG). Incident CHD was defined as sudden cardiac death, definite or probably MI, or coronary revascularization procedures (CABG or PCI). Genotyping was performed with the Illumina Human 1Mv1_C chip at deCODE Genetics in Reykjavik, Iceland; samples were excluded due to: 1) gender discrepancies, 2) Mendelian inconsistency rate>5%, or 3) ancestry outliers from any of the first 10 principal components from EIGENSTRAT. SNPs missing chromosomal location, SNPs with HWE p<10^−8^, monomorphic SNPs, and SNPs with call rate <90% or with strand issues were excluded from analysis. Imputation to the 1000 G Phase I Integrated Release Version 3 [March 2012] was performed with IMPUTE2. Data were analyzed using R v2.15.1 (survival package), under an additive model.

The CHARGE Consortium performed a GWA analysis of incident MI and CHD in 24,024 participants of European ancestry from five prospective cohort studies (ARIC, CHS, Framingham Heart Study and Rotterdam study) including 1,570 cases of MI, and 2,406 cases of CHD. CHD was defined as fatal and non-fatal MI and CVD mortality. Only first event was included.

### Statistical analysis

Cox proportional hazard models were implemented in each discovery and replication study, after excluding individuals with history of CHD at baseline. We used additive genetic models (allele dosage) in sex-specific analyses and adjusted for age at the first examination visit, center or geographic region and measures of population stratification using study-specific principal components. Study- specific log(hazard ratio) estimates were combined using inverse-weighted variance meta-analyses methods [30]. We used a minor allele frequency of 0.01 for filtering SNPs during meta-analysis. We also tested for the evidence of between-study heterogeneity [31] using an alpha  = 0.05. Summary estimates (hazard ratio, HR) and 95% confidence intervals (CI) are reported.

#### Fine mapping of known loci

We identified approximately 50 regions of interest based on previously reported loci in European ancestry and Asians by querying the genome catalog for all variants achieving a genome wide threshold of *P*<5.0×10^−8^ for CHD associations (http://www.genome.gov/gwastudies/). These included new CHD loci published after the MetaboChip array was designed; of these only 44 loci had SNPs genotyped on the MetaboChip array. We first evaluated the association of previously published SNPs with incident CHD in the discovery sample of African Americans. We then searched for the lowest p-value SNP in the region (boundaries defined by 250 kb at each side of leading SNP or proxy) and examined the LD of the SNP with the published SNP using LD statistics estimated in the HapMap 1000 G CEU samples (representing European ancestry) and in the ARIC/WHI African American data (representing African American LD). We considered low LD as r^2^<0.3. We also examined if the direction of the effect for the coded allele was the same between the original publication and the data reported here. For the fine mapping analyses, we considered a Bonferroni corrected p-value adjusted for the number of tested SNPs at each region (see **Table 1**).

**Table 1:** Associations of validated CHD loci with incident coronary heart disease in African Americans.

					Metabochip findings							LD with published SNP	
Locus	Nearby Gene	Lead Published SNP or proxy	Coded Allele	Coded allele freq AA	*P*	Hazard ratio	Pub OddRatio	Most significant SNP on MetaboChip	Coded allele freqAA	*P*	N SNPs/*P* for threshold	CEU.r2	AA.r2
1p13.3	*SORT1*	rs599839	A	0.29	2.7E-03	1.28	1.29	rs583104	0.27	2.2E-04	916/5.5E-05	1.00	0.997
1q41	*MIA3*	rs17465637	C	0.74	0.32	1.09	1.14	rs112045392	0.05	4.6E-04	548/9.1E-05	NA	0.02
13q34	*COL4A1-COL4A2*	rs4773144	G	0.62	0.96	0.99	1.07	rs12855875	0.07	8.8E-03	1176/4.3E-05	<0.01	0.02
1p32.2	*PPAP2B*	rs17114036	A	NA	NA	NA	1.09	rs112429198	0.02	2.2E-03	357/1.4E-04	<0.01	<0.01
1p32.3	*PCSK9*	rs11206510	A	0.85	0.17	1.08	1.06	rs572512	0.22	1.4E-03	147/3.4E-04	0.04	0.02
1q24.3	*VAMPS*	rs1561198	A	0.69	0.39	0.90	1.05				29/1.7E-03		
1q21.3	*IL6R*	rs4845625	T	0.70	0.92	1.03	1.06				28/1.8E-03		
2q33.1	*WDR12*	rs6725887	C	0.97	0.03	1.49	1.12	rs115344174	0.98	8.8E-04	933/5.4E-05	NA	<0.01
2p24.1	*APOB*	rs515135	G	0.52	0.67	1.06	1.07	rs12720789	0.03	4.8E-04	1354/3.7E-05	NA	<0.01
2q22.3	*ZEB2*	rs2252641	G	0.80	0.54	1.08	1.06				15/3.3E-03		
2p21	*ABCG5*	rs6544713	T	0.79	0.70	1.05	1.06	rs4953032	0.66	2.3E-03	1024/4.9E-05	0.01	<0.01
3q22.3	*MRAS*	rs9818870	A	0.09	0.58	0.99	1.07	rs79466163	0.96	5.9E-03	745/6.7E-05	NA	<0.01
4q32.1	*GUCY1A3*	rs7692387	G	0.92	0.33	1.14	1.08				49/1.0E-03		
4q31.22	*EDNRA*	rs1878406	T	0.18	0.39	0.97	1.10				57/8.8E-04		
5q31.1	*SLC22A4*	rs273909	C	NA	NA	1.07	1.07				53/9.4E-04		
6p21.31	*ANKS1A*	rs2077750*	A	0.04	0.28	1.11	NA				81/6.2E-04		
6p21.2	*KCNK5*	rs10947789	T	0.95	0.81	1.02	1.07	rs9349112		6.7E-03	51/9.8E-04	<0.01	<0.01
6q26	*PLG*	rs4252120	T	0.82	0.82	1.05	1.07				1089/4.6E-05		
6p24.1	*PHACTR1*	rs9369640	A	0.91	0.79	1.13	1.09				1910/2.6E-05		
6q25.3	*SLC22A3- LPA*	rs3798220	C	NA	NA	NA	NA	rs61131294	0.84	7.7E-03	1420/3.5E-05	NA	NA
6q23.2	*TCF21*	rs12190287	C	0.10	0.60	0.97	1.07	rs328455	0.98	3.7E-03	470/1.1E-04	0.08	<0.01
7q32.2	*ZC3HC1*	rs11556924	C	0.09	0.95	1.02	1.09				153/3.3E-04		
7p21.1	*HDAC9*	rs2023938	G	0.83	0.58	0.95	1.08				33/1.5E-03		
8q24.13	*TRIB1*	rs2954029	A	0.66	0.74	1.09	1.06	rs71516794	0.05	1.5E-04	449/1.1E-04	<0.01	<0.01
8p21.3	*LPL*	rs264	G	0.87	0.66	0.97	1.11	rs73667448	0.96	2.0E-03	1147/4.4E-05	NA	<0.01
9p21.3	*CDKN2A,2B*	rs2891168†	A	0.79	0.21	0.92	NA	rs17694555	0.98	2.5E-04	688/7.3E-05	0.05	0.02
9q34.2	*ABO*	rs579459	C	0.86	0.03	1.19	1.07				1195/4.2E-05		
10q24.32	*CYP17A1*	rs12413409	G	0.06	0.39	1.05	1.10	rs80236706	0.06	6.9E-03	1624/3.1E-05	<0.01	<0.01
10q11.21	*CXCL12*	rs1746048	C	0.45	0.07	1.16	1.17				1754/2.9E-05		
		rs501120	A	0.59	0.13	1.14	1.07				1753/2.9E-05		
10p11.23	KIAA1462	rs2505083	G	0.10	0.25	0.93	1.06				33/1.5E-03		
10q23.2-q23.3	*LIPA*	rs1412444	T	NA	NA	NA	1.09				41/1.2E-03		
		rs2246833	A	0.41	0.42	1.03	1.06				40/1.3E-03		
11q22.3	*PDGFD*	rs974819	A	0.50	0.55	0.997	1.07				27/1.9E-03		
11q23.3	*ZNF259-APOA5*	rs964184	C	NA	NA	NA	1.09	11216103	0.02	2.0E-03	657/7.6E-05	NA	NA
12q24.12	*SH2B3*	rs3184504	A	0.09	0.35	0.91	1.07				1784/2.8E-05		
13q12.3	*FLT1*	rs9319428	A	0.30	0.39	0.96	1.06				25/2.0E-03		
14q32.2	*HHIPL1*	rs2895811	C	0.76	0.23	0.996	1.06	rs4445835	0.61	5.6E-03	28/1.8E-03	0.02	<0.01
15q26.1	*FURIN*	rs17514846	A	0.81	0.79	0.95	1.07	rs34050628	0.03	6.5E-03	558/9.0E-05	0.21	0.11
15q25.1	*ADAMTS7*	rs3825807	A	NA	NA	NA	1.07				671/7.5E-05		
17q21.32	*UBE2Z*	rs15563	C	0.85	0.85	1.01	1.04				54/9.3E-04		
17p11.2	*RASD1*	rs12936587	G	0.31	0.12	1.03	1.06				33/1.5E-03		
17p13.3	*SMG6*	rs216172	C	0.01	0.82	0.90	1.04				31/1.6E-03		
19p13.2	*LDLR*	rs1122608	G	0.05	0.93	0.99	1.10	rs3786721	0.35	3.2E-03	158/3.2E-04	0.11	0.01
19q13.32	*APOE-APOC1*	rs2075650	G	0.88	0.33	0.85	1.11				179/2.8E-04		
21q22.11	*MRPS6-KCNE2*	rs9982601	A	0.24	0.09	1.11	1.13				382/1.3E-04		

Abbreviations: LD, linkage disequilibrium; SNP, single nucleotide polymorphism; AA, African Americans from PAGE studies; NA, not available. Only SNPs with minor allele frequency>0.01 that passed quality control are included. Linkage disequilibrium in African Americans was calculated in the ARIC study and allele frequencies from the ARIC and WHI genotyped data. Thresholds for significant associations for SNPs with LD r^2^<0.3 with published SNP were based on the number of SNPs in each region.

^*^proxy of rs1760994,

† proxy of rs4977574. Between-study heterogeneity was not significant (p<0.05) except for SNPs rs9982601 (p = 0.009) and rs10947789 (p = 0.02). Hazard ratios are for incident CHD associations from this study; odds ratio are from published results from the publication from Deloukas, P., S. Kanoni, et al. (2013). "Large-scale association analysis identifies new risk loci for coronary artery disease." Nature Genetics. 45(1): 25–33.

#### Analyses for novel loci

For the discovery of novel CHD loci in PAGE African Americans using the MetaboChip, we applied a genome wide threshold of 2.8×10^−7^ for 163,270 SNPs tested based on a Bonferroni correction. SNPs with a *P*<10^−5^ were carried forward for replication in additional African American samples and in individuals of European ancestry from the CHARGE Consortium. Replication was defined as a Bonferroni corrected *P*<0.05 in the replication sample or a combined meta-analysis discovery and replication *P* lower than the discovery *P*.

To functionally annotate validated CHD-associated SNPs we used HaploReg, which integrates data from the ENCyclopedia Of DNA Elements (ENCODE) Project Consortium [32]–[34] resources as well as other resources such as published expression QTL studies. For CHD-associated SNPs that occur within annotated 3′ untranslated regions (3′ UTRs), we additionally examined the possibility of microRNA target site creation/disruption by mining a recently published database of genetic variants in candidate miRNA target sites [35].

## Results and Discussion

The characteristics of the PAGE African American participants are shown in **S1 Table**. After QC, a total of 546 incident CHD events among the 8,201 African American individuals with MetaboChip genotyping were available. The average age at intake into the cohort was 54 years and 61 years in ARIC and the WHI respectively, and their median follow-up was 18 years and 12 years.

**Figure 1:**
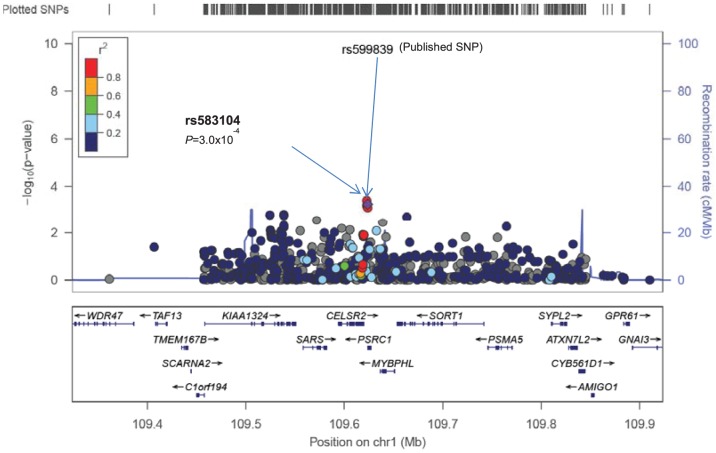
Regional plots of validated loci showing replication of the published SNP or proxy (a) or novel SNPs in known CHD loci (b, c). The X-axis shows chromosomal positions and genes, the left Y-axis shows the –log10P-values and the right Y-axis shows the recombination rate across the region using HapMap CEU linkage disequilibrium. Plotted SNP is the published SNP and is marked by a purple diamond and an arrow.

Analyses of time to first CHD event were performed within each study and within sex-strata. Study-specific genomic control values were <1.0 after filtering based on minor allele frequency of 0.01, indicating little evidence for global population stratification (lambdas 0.93, 0.93 and 0.86 for ARIC women, ARIC men, and WHI women, respectively). We first examined the evidence for replication and fine mapping of previously published CHD loci for incident CHD in African Americans (**Table 1**). The best evidence for SNP replication was at the *SORT1* locus, where rs583104 (minor allele frequency [MAF]  = 0.27, *P* = 2.2×10^−4^) is in complete LD with rs599839 (r^2^ = 1.0 in HapMap CEU, *P* = 2.7×10^−3^), the SNP described in the European ancestry GWAS [10], [13] (**Fig. 1**). Each copy of the rs599839 A allele was associated with a 18% increased hazard of CHD. All SNPs in 1000 Genomes Project (phase 3) HapMap CEU samples with r^2^ with rs599839 greater than 0.25 lie within a 130 kb window centered at rs599839 (displayed in **Fig. 1**). Of the 14 SNPs in the 1000 Genomes Project (phase 3) with r^2^ in CEU samples greater than 0.5 with rs599839, 11 are on the Metabochip. Only rs3902354 (r^2^ = 0.54 in CEU with rs599839), rs4970836 (r^2^ = 0.95), and rs4970837 (r^2^ = 0.56) are not on the Metabochip. Each of these 3 SNPs, however, is in high LD (r^2^>0.95) in CEU with SNPs on the Metabochip. There was evidence for fine-mapping at this locus in African Americans, as only 3 SNPs (rs583104, rs602633 and rs1277930, encompassing a 0.8 Mbp region) were correlated with rs599839 (r^2^>0.5) in African Americans compared to 11 SNPs (7.3 Mbp) in HapMap CEU 1000 G samples. The previously described SNP rs12740374 (MAF  = 0.25) was not associated with incident CHD (*P* = 0.22) and it was in low LD with rs599839 (r^2^ = 0.14) in our data. None of the remaining published SNPs reached significance after adjusting for multiple testing, although 22 of 39 published SNPs available in our data, the estimates had the same direction of effect as reported in studies of individuals of European ancestry (**Table 1**).

We then searched for additional SNPs associated with incident CHD in each region and identified twenty-two loci that had at least one other SNP not in LD with the published SNP (r^2^<0.3) with low p-value in the region (**Table 1**). These SNPs included low frequency and common variants, and the associations did not replicate in additional African American participants of WHI-SHARe and GeneStar, or in European ancestry samples from the publicly available CARDIoGRAM plusC4D meta-analysis (**S2 Table** **)**.

**Figure 2:**
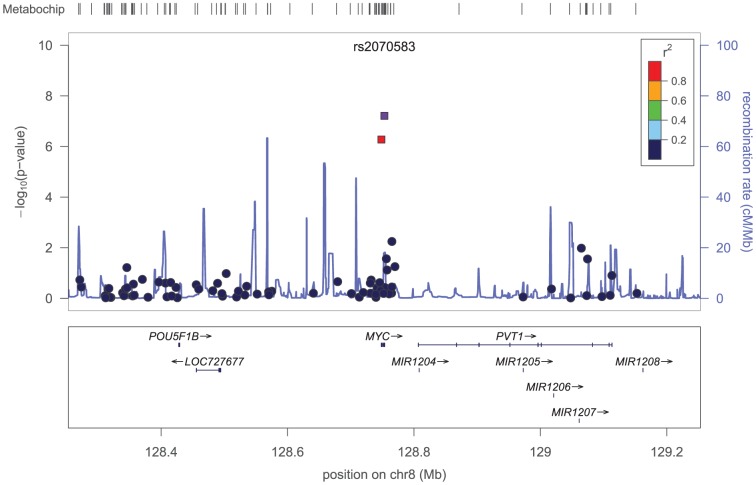
Regional plots of the *MYC* loci showing two SNPs and their LD. The X-axis shows chromosomal positions and genes, the left Y-axis shows the –log10P-values and the right Y-axis shows the recombination rate across the region using HapMap CEU linkage disequilibrium. Plotted SNPs are marked by arrows.

We also evaluated the evidence for association of novel loci with incident CHD in PAGE African American genotyped discovery samples. Meta-analyses of the genotyped estimates from each study showed three SNPs, two within *MYC* and one near to *ADRA1A*, associated with incident CHD and eligible for replication based on a pre-specified threshold of *P*<10^−5^ (**Table 2**). SNP rs2070583 (MAF 0.02), located at the 3′UTR of the *MYC* gene, reached the Bonferroni-corrected genome wide significant threshold for the MetaboChip array (*P*<2.8×10^−7^). This SNP was in strong LD with another SNP in the *MYC* gene (rs4645948) (r^2^>0.90, HapMap YRI) (**Fig. 2**). These SNPs and an intergenic SNP near *ADRA1A* were carried forward for replication in independent samples consisting of additional 8,059 African American individuals (577 CHD events) with 1000 G imputed data (**Table 2**). The replication samples consisted of two samples of slightly older individuals (mean age 73.3 years, standard deviation 2.9) than in PAGE samples free of CHD at baseline (61.8 years, standard deviation 7.1) and an early CHD sample (**S1 Table**). rs2070583 had a low imputation quality for a low frequency variant in the available imputed samples from WHI and HealthABC data (rsq  = 0.73 and 0.53, respectively) and associations did not replicate for this SNP. The other *MYC* SNP, rs4645948, and the SNP nearby the *ADRA1A* gene, rs1965328, also did not replicate in the imputed African American samples. Allelic frequencies of *MYC* SNPs in European ancestry populations are similar to those in the African American population. We therefore pursued replication in an additional sample of European ancestry subjects with GWA data in the CHARGE Consortium but found no evidence for replication of associations in these samples (**Table 1**).

**Table 2:** Associations at novel loci for incident CHD in African Americans: discovery and replication.

				Discovery			Replication						
				African Americans*			African Americans†		European ancestry#		Discovery and replicationAfrican Americans		
Nearby gene	SNP	Allele	MAF	Event/Total	HR (95% CI)	*P*	Event/Total	*P*	Event/Total	*P*	Beta (se)	*P*	Event/Total
*MYC*	rs2070583	A/G	0.02	546/8,201	2.47 (1.77, 3.44)	8.1×10^−8^	577/8,059	0.30	2,405/24,024	0.79	−0.6594 (0.1324)	6.3×10^−7^	17,513
*MYC*	rs4645948	A/G	0.02	546/8,198	2.46 (1.74, 3.48)	3.8×10^−7^	577/8,059	0.48	2,405/24,024	0.77	0.6259 (0.1404)	8.2×10^−6^	16,257
*ADRA1A*	rs1965328	A/G	0.52	546/8,199	1.32 (1.17, 1.50)	7.9×10^−6^	577/8,059	0.52	2,405/24,024	0.07	0.1166 (0.0437)	7.7×10^−3^	16,258

CI, confidence interval; HR, hazard ratio; MAF, minor allele frequency; N, number; SNP, single nucleotide polymorphism. MAF for SNPs in HapMap CEU samples are: rs2070583 G allele  = 0.009, rs4645948 T allele  = 0.027 and rs1965328 A allele  = 0.265.

*P* for between-study heterogeneity was not significant. SNPs were eligible for replication if *P*<1.0×10^−5^. The array wide significant threshold is *P*<2.8×10^−7^. Replication was considered a P<0.05 in replication samples and/or a *P* less than discovery in the combined discovery and replication samples. Note CHD events do not include procedures.

* Atherosclerosis Risk in Communities study and Women's Health Initiative study; † Additional samples from Women's Health Initiative, GeneSTAR and Health ABC studies; #CHARGE Consortium.

Finally, we identified several SNPs located in regions with some evidence of regulatory function based on HaploReg analysis (**S3 Table**).

In this prospective genetic study of CHD in African American individuals using a high density genotyping array, we replicated and fine-mapped one locus (*SORT1*), which has not achieved significance in previous African American studies for CHD or coronary artery calcification [19], [21], [36], [37]. In the *SORT1* locus, the SNP with lowest p-value in the region (rs583104) was in LD with the published SNP (rs599839). As previously reported, these SNPs occur within a genomic region that is bound by numerous transcription factors, and is predicted to alter the affinity of binding for some of those transcription factors (e.g., PU.1). Of interest, the rs599839 risk allele showed same direction of effect but larger magnitude of risk for incident CHD (hazard ratio 1.8) compared to published cross-sectional case-control studies (odds ratio of 1.1 to 1.3) [6], [10]. Our study shows consistency in the direction of effect for additional 22 published SNPs, although associations did not reach the significance threshold.

Our novel finding at the *MYC* gene is for two low frequency variants (MAF  = 0.02) including rs2070583, a SNP located on the 3′ UTR gene region, which showed 2.5-fold higher' hazard of CHD per copy of the A allele, although the associations remained suggestive given the lack of replication of findings. c-myc protein is a transcription factor with function in cell cycle progression, apoptosis and cellular transformation [38], recently shown to relate to coronary vascular development and remodeling [39]. There is additional biological support for the relevance of this gene to CVD. For example, c*-myc* +/- heterozygote mice display decreased coronary vasculature when compared to wild type (WT) littermates [39] and mice with induced c-myc expression in cardiomyocytes develop hypertrophic cardiomyopathy, ventricular dysfunction and heart failure [40]. However, we were unable to validate this locus in our replicating samples. This association is unlikely to be identified previously due to the low frequency of these variants (MAF  = 0.02) and low coverage of this gene in other genome arrays. This may explain at least in part our failure to replicate the results in African Americans and individual of European ancestry in which the coverage of the *MYC* gene is poor. In addition, as compared to genotyped data used for the discovery, the replication samples consisted of imputed data with varying quality of imputation. Therefore, we cannot exclude false positive results for this locus, which should be further investigated in studies using exome data.

This is the largest genetic study to use adjudicated incident clinical events in African Americans. In contrast to most prior genetic studies of CHD, our analysis was restricted to harmonized, adjudicated incident clinical events among individuals without prior history of CHD. This study differs from prior studies by using prospective incident data instead of a case-control design. In addition, we used a high density genotyping array which included common and low frequency genotyped variants from 44 published CHD loci. However, our study is limited by the available samples and the coverage of the genotyped array. We have reasonable power to detect HRs of 1.4 and greater for SNPs with allele frequencies greater than 0.25 and HRs of 1.6 for SNP with allele frequency between 0.05 and 0.25 (S2 File). There is some overlap of individuals in the ARIC study from the previously reported incident CHD paper by Barbalic *et al*, which examined 2,905 participants as compared to 3,204 used in our analysis. This prior publication studied only GWA genotyped data, with low coverage on several loci described in our study.

In summary, in loci previously reported to be associated with CHD, a published SNP (rs599839, *SORT1*) was associated with incident CHD in African American individuals. We also provide evidence for fine-mapping at this locus, and identified an array wide association for a SNP in *MYC*, a gene with some biology plausibility for CHD traits, which will require further validation in additional samples. This study extends the genetic findings of associations of validated CHD to African Americans and for incident CHD events.

## Supporting Information

S1 Table **Demographic characteristics of the African American individuals from studies with incident CHD events.**
(DOCX)

S2 Table **Associations of secondary signals in validated CHD loci with incident coronary heart disease in African Americans.**
(DOCX)

S3 Table **Bioinformatic functional annotation of SNPs.**
(DOCX)

S1 File **Study descriptions.**
(DOCX)

S2 File **Power analysis.**
(DOCX)
