# Supplementary material for: Prospective Associations of Coronary Heart Disease Loci in African Americans Using the MetaboChip: The PAGE Study
Source: PLoS One. 2014 Dec 26;9(12):e113203. doi: 10.1371/journal.pone.0113203 (PMC4277270; doi:10.1371/journal.pone.0113203)
Supplement: S1 Table — Demographic characteristics of the African American individuals from studies with incident CHD events. (DOCX) [file pone.0113203.s001.docx]

| **Table S1.** Demographic characteristics of the African American individuals from studies with incident CHD events | | | | | | | |
| --- | --- | --- | --- | --- | --- | --- | --- |
|  | **Discovery** | | | **African American Replication** | | | |
|  | **ARIC Men** | **ARIC Women** | **WHI** | **WHI-SHARe** | **HealthABC Men** | **HealthABC Women** | **GeneStar*** |
| Mean age (SD), years | 53.6 (6.0) | 53.3 (5.7) | 60.8 (7.1) | 61.8 (7.1) | 73.3 (2.8) | 73.3 (2.9) | 42.1 (10.3) |
| Prevalent CHD | 0 | 0 | 0 | 0 | 0 | 0 | 0 |
| No. Cases/Total No. | 174/1,172 | 192/2,032 | 164/4,997 | 310/6105 | 111/368 | 112/527 | 44/1059 |
| Median follow up, years | 18.3 | 18.6 | 9.9 | 7.9 | 5.8 | 6.5 | 6.0 |
| Follow up year | 2007 | 2007 | 2009 | 2009 | 2011 | 2011 | 2011 |

CHD, coronary heart disease; SD, standard deviation; No., number Individuals with prevalence CVD at baseline were excluded Note: discovery samples are genotyped and replication samples are from imputed data, see methods. * 62.7% are women. Data were analyzed using R v2.15.1 (survival package), under an additive model.
