# Supplementary material for: Prospective Associations of Coronary Heart Disease Loci in African Americans Using the MetaboChip: The PAGE Study
Source: PLoS One. 2014 Dec 26;9(12):e113203. doi: 10.1371/journal.pone.0113203 (PMC4277270; doi:10.1371/journal.pone.0113203)
Supplement: S2 Table — Associations of secondary signals in validated CHD loci with incident coronary heart disease in African Americans. (DOCX) [file pone.0113203.s002.docx]

**Table S2.** Associations of secondary signals in validated CHD loci with incident coronary heart disease in African Americans

|  |  | Coded Allele | | | AA Discovery | | Replication p-values  AA samples EA samples | | | |
| --- | --- | --- | --- | --- | --- | --- | --- | --- | --- | --- |
| Locus | SNP | All | Freq AA | Freq EA | HR | *P* | GeneStar | WHI-SHARe | Meta-analysis | CARDIoGRAM plusC4D* |
| 1q41- *MIA3* | rs112045392 | A | 0.05 | NA | 1.55 | 4.6E-04 | 0.09 | 0.16 | 0.70 |  |
| 1p32.2 - *PPAP2B* | rs112429198 | A | 0.02 | 0.05 | 1.85 | 2.2E-03 | 0.54 | 0.93 | 0.95 |  |
| 1p32.3 - *PCSK9* | rs2317949 | A | 0.28 | 0.19 | 1.24 | 1.4E-03 | 0.97 | 0.72 | 0.84 | 0.59 |
| 2q33.1- *WDR12* | rs115344174 | G | 0.02 | NA | 1.80 | 8.8E-04 | 0.49 | 0.85 | 0.64 |  |
| 2p24.1 - *APOB* | rs12720789 | A | 0.02 | 0.004 | 2.09 | 4.8E-04 | 7.4E-09 | 0.55 | 0.67 |  |
| 2p21- *ABCG5* | rs4953032 | A | 0.67 | 0.58 | 1.24 | 2.3E-03 | 0.46 | 0.26 | 0.77 | 0.73 |
| 3q22.3 - *MRAS* | rs79466163 | G | 0.04 | NA | 1.29 | 5.9E-03 | 0.62 | 0.02 | 0.25 |  |
| 6p21.2 - *KCNK5* | rs2758888 | G | 0.45 | 0.43 | 1.18 | 6.7E-03 | 0.91 | 0.76 | 0.81 | 0.90 |
| 6q25.3- *SLC22A3* | rs61131294 | G | 0.17 | 0.18 | 1.11 | 7.7E-03 | 0.08 | 7.2E-03 | 0.36 |  |
| 6q23.2 - *TCF21* | rs328455 | G | 0.02 | 0.11 | 1.77 | 3.7E-03 | 0.76 | 0.38 | 0.63 | 0.16 |
| 8q24.13 - *TRIB1* | rs71516794 | A | 0.05 | NA | 1.57 | 1.5E-04 | 0.82 | 0.69 | 0.89 |  |
| 8p21.3 - *LPL* | rs73667448 | C | 0.04 | NA | 1.52 | 2.0E-03 | 0.83 | 0.10 | 0.27 |  |
| 9p21.3 - *CDKN2* | rs7021012 | G | 0.19 | 0.47 | 1.32 | 2.5E-04 | 0.47 | 0.28 | 0.30 | 0.25 |
| 10q24.32 - *CYP17A1* | rs7094325 | C | 0.40 | 0.32 | 1.23 | 8.4E-04 | 0.81 | 0.92 | 0.87 |  |
| 11q23.3 - *ZNF259* | rs11216103 | A | 0.02 | 0.04 | 1.78 | 2.0E-03 | 0.93 | 0.35 | 0.53 | 0.03 |
| 13q34- *Col4A1/4A2* | rs12855875 | G | 0.07 | 0.19 | 1.36 | 8.8E-03 | 0.02 | 0.30 | 0.99 | 0.66 |
| 14q32.2 - *HHIPL1* | rs12884657 | G | 0.18 | 0.32 | 1.24 | 5.6E-03 | 0.04 | 0.21 | 0.65 | 0.12 |
| 15q26.1 - *FURIN* | rs34050628 | A | 0.02 | 0.06 | 1.67 | 6.5E-03 | 0.68 | 0.44 | 0.79 |  |
| 19p13.2 - *LDLR* | rs3786721 | A | 0.34 | 0.48 | 1.21 | 3.2E-03 | 0.76 | 0.22 | 0.58 | 0.04 |

Replication in the CARDIoGRAMplusC4D Consortium was done using publicly available data (<http://www.cardiogramplusc4d.org/downloads/>).

Abbreviations: ALL, allele; SNP, single nucleotide polymorphism; AA, African Americans from PAGE studies; EA, European ancestry from HapMap CEU; NA, not available; *P*, p-value. Only SNPs with minor allele frequency > 0.01 that passed quality control are included.

Data were analyzed using R v2.15.1 (survival package), under an additive model.
