# Supplementary material for: Prospective Associations of Coronary Heart Disease Loci in African Americans Using the MetaboChip: The PAGE Study
Source: PLoS One. 2014 Dec 26;9(12):e113203. doi: 10.1371/journal.pone.0113203 (PMC4277270; doi:10.1371/journal.pone.0113203)
Supplement: S3 Table — Bioinformatic functional annotation of SNPs. (DOCX) [file pone.0113203.s003.docx]

**Table S3.** Bioinformatic functional annotation of SNPs in high LD (r^2^>0.8 in AFR) identified in validated and new loci for incident coronary heart disease. Data obtained from HaploReg at www.broadinstitute.org/mammals/haploreg/haploreg.php


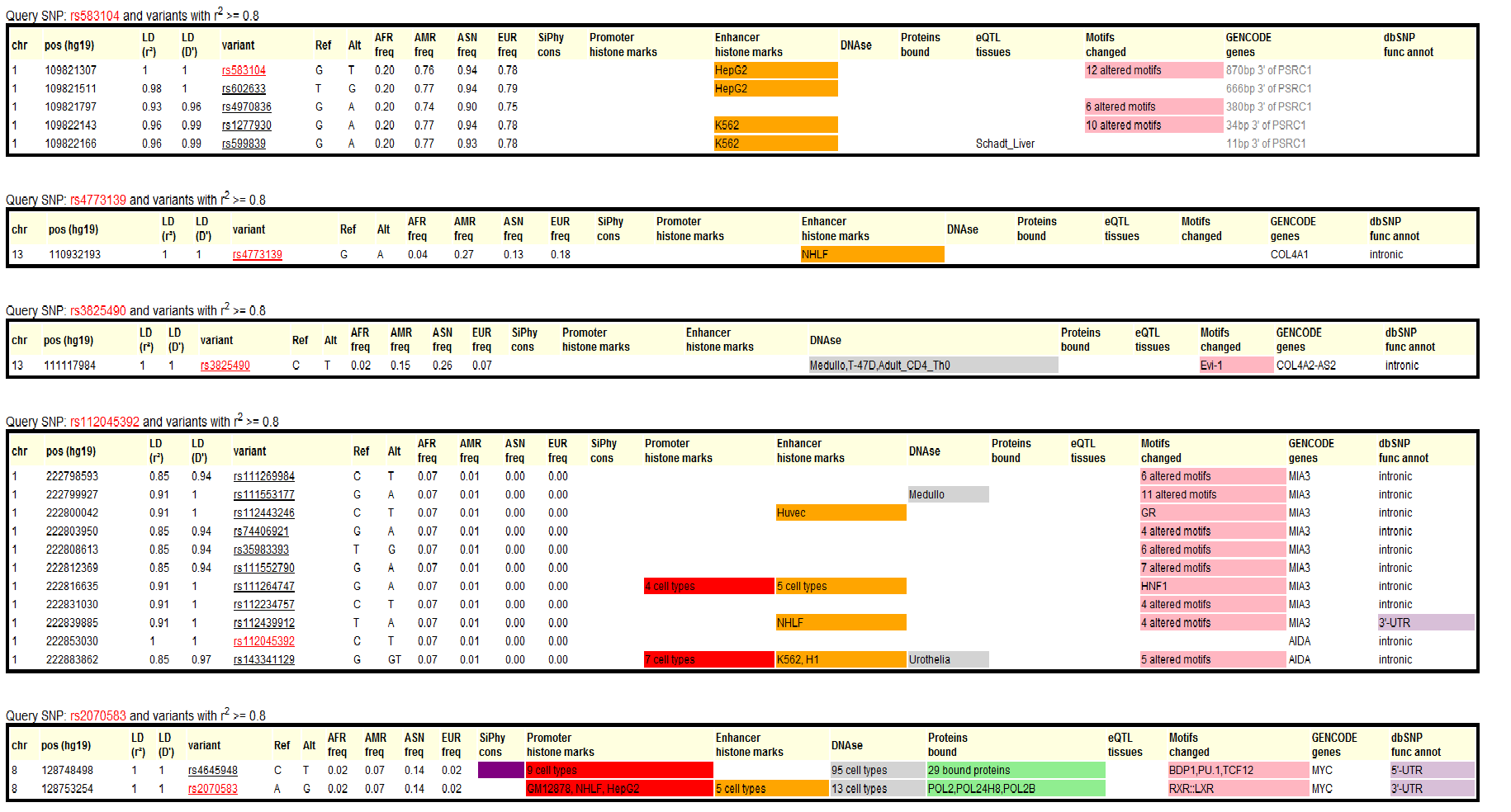


ed using R v2.15.1 (survival package), under an additive model.
