# Supplementary material for: Prospective Associations of Coronary Heart Disease Loci in African Americans Using the MetaboChip: The PAGE Study
Source: PLoS One. 2014 Dec 26;9(12):e113203. doi: 10.1371/journal.pone.0113203 (PMC4277270; doi:10.1371/journal.pone.0113203)
Supplement: S1 File — Study descriptions. (DOCX) [file pone.0113203.s004.docx]

**Study Descriptions**

**Discovery study samples**

The ARIC study is a multi-center cohort and community surveillance investigation in predominantly bi-racial populations (white and African Americans)[[23](#_ENREF_23)]. ARIC recruited 15,792 individuals (of which 4,266 are African Americans) aged 45-64 years from four communities in Forsyth County, N.C., Jackson, M.S., Minneapolis, M.N., and Washington County, M.D. for a baseline examination in 1987-1989, with four follow-up examinations. Annual follow-up and community surveillance identified CHD events (defined below) which are then classified by an expert panel of physicians based on review of hospital records, death certificates and interviews of next of kin [[23](#_ENREF_23)]. All participants included in these analyses have given consent for genetic studies and data sharing.

WHI is a prospective study investigating post-menopausal women’s health in the U.S [[24](#_ENREF_24)]. A total of 161, 838 women aged 50–79 years old were recruited from 40 US clinical centers between 1993 and 1998 to participate in an observational study (OS) and in three clinical trials (CT). Annual (OS) and semi-annual (CT) follow-up identified self-reported events which were then classified by an expert panel of physicians based on review of hospital records, death certificates and interviews of next of kin [[25](#_ENREF_25)].

**Replication studies description**

HealthABC is a prospective cohort study of 3,075 community-dwelling men and women living in Memphis, Tennessee, USA and Pittsburgh, Pennsylvania, USA, aged 70–79 years at recruitment in 1997 [[27](#_ENREF_27)], of which 42% were African Americans. A random sample of European ancestry and African American Medicare-eligible elders, within designated zip code areas, were contacted. Eligibility included reporting no difficulty with activities of daily living, walking a quarter of a mile, or climbing ten steps without resting. All eligible participants signed a written informed consent, approved by the IRBs at the clinical sites. This study was approved by the IRBs of the clinical sites and the coordinating center (University of California, San Francisco). Individuals with prevalent CHD defined as MI, angina, bypass surgery, or percutaneous transluminal coronary angioplasty (PTCA) procedures or diagnoses reported to the Centers for Medicare and Medicaid Services in the five years prior to baseline, were excluded. Incident CHD was defined as adjudicated event of MI, hospitalization for angina, or adjudicated CHD death in participants who did not have prevalent CHD. The sample included 895 participants and 223 events. Follow-up is through December 31, 2011, for a median of 11.0 years. Genotyping was done on Illumina 1M microarrays and the GWAS data underwent standard QC procedures including evaluation of genotype and self-reported sex concordance, sample call rate of 97% or higher, exclusion of heterogeneity or homozygosity outliers, relatedness (IBD > 0.125 were excluded) and ancestry outliers.  All SNPs were filtered for inclusion based on a minimum call rate of 97%, HWE > 10^-6^, minor allele frequency > 1% and a missingness by haplotype p-value > 10^-5^.  Samples were imputed to the most recent multiethnic panel of the 1000G reference haplotypes (phase 1 alpha version 3) using MACH and minimac. Analyses were carried out using R v2.14.2, with the first ten principal components in the African American population as a means of controlling for population substructure.

The Genetic Study of Atherosclerosis Risk study (GeneSTAR) study identified probands with documented premature (age <60 years) CHD events including MI, coronary artery bypass surgery (CABG), percutaneous coronary intervention (PCI), or ≥ 50% stenosis in one or more vessels confirmed on coronary angiography with or without angina symptoms at the time of hospitalization in one of 10 Baltimore, Maryland hospitals. Their apparently healthy siblings < 60 years of age and free of known CHD were recruited and screened from 1983 to 2007, and apparently healthy offspring of the probands or siblings were recruited and screened from 2003-2007. Participants were excluded from the study for systemic autoimmune disease, chest radiation exposure, any life-threatening disease (AIDS or advanced cancer), or chronic glucocorticosteroid therapy. Participants completed a standardized health status and cardiovascular disease event questionnaire at approximately five-year intervals after their baseline visit with trained telephone interviewers between 1992 and 2012. For deceased siblings, proxy interviews were completed with the next of kin, and death certificates were obtained. Medical records were then obtained for all reports of a CHD event, any possibly related diagnosis, diagnostic procedure (including exercise tests, thallium imaging, or coronary angiography) or therapeutic procedure (including PCI or CABG). Incident CHD was defined as sudden cardiac death, definite or probably MI, or coronary revascularization procedures (CABG or PCI). Genotyping was performed with the Illumina Human 1Mv1_C chip at deCODE Genetics in Reykjavik, Iceland; samples were excluded due to: 1) gender discrepancies, 2) Mendelian inconsistency rate > 5%, or 3) ancestry outliers from any of the first 10 principal components from EIGENSTRAT. SNPs missing chromosomal location, SNPs with HWE p< 10^-8^, monomorphic SNPs, and SNPs with call rate < 90% or with strand issues were excluded from analysis. Imputation to the 1000G Phase I Integrated Release Version 3 [March 2012] was performed with IMPUTE2. Data were analyzed using R v2.15.1 (survival package), under an additive model.
