# Supplementary material for: Prospective Associations of Coronary Heart Disease Loci in African Americans Using the MetaboChip: The PAGE Study
Source: PLoS One. 2014 Dec 26;9(12):e113203. doi: 10.1371/journal.pone.0113203 (PMC4277270; doi:10.1371/journal.pone.0113203)
Supplement: S2 File — Power analysis. (DOCX) [file pone.0113203.s005.docx]

**Power analysis**

Power was estimated using Quanto (v.1.2.4), assuming alpha=0.05, 546 CHD cases, 7,655 controls, disease prevalence =0.08. For a given allele frequency and hazard ratio, the power to detect an association is given below:

| Coded Allele Frequency | Hazard Ratio | Power |
| --- | --- | --- |
| 0.01 | 1.2 | 0.09 |
|  | 1.4 | 0.21 |
|  | 1.6 | 0.39 |
|  | 1.8 | 0.58 |
| 0.05 | 1.2 | 0.25 |
|  | 1.4 | 0.68 |
|  | 1.6 | 0.94 |
|  | 1.8 | >0.95 |
| 0.25 | 1.2 | 0.54 |
|  | 1.4 | >0.95 |
|  | 1.6 | >0.95 |
|  | 1.8 | >0.95 |
| 0.40 | 1.2 | 0.49 |
|  | 1.4 | 0.94 |
|  | 1.6 | >0.95 |
|  | 1.8 | >0.95 |
| 0.50 | 1.2 | 0.41 |
|  | 1.4 | 0.88 |
|  | 1.6 | >0.95 |
|  | 1.8 | >0.95 |

In general, we have reasonable power to detect HRs of 1.4 and greater for SNPs with coded allele frequencies greater than 0.25. We have low power to detect associations for low frequency SNPs (allele frequency<0.05), given an expected hazard ratio range of 1.2-1.8.

**Reference:**

Gauderman WJ, Morrison JM. QUANTO 1.1: A computer program for power and sample size calculations for genetic-epidemiology studies, http://hydra.usc.edu/gxe, 2006.

ere analyzed using R v2.15.1 (survival package), under an additive model.
